# Marine algae as emerging therapeutics in lung health

**DOI:** 10.17179/excli2026-9388

**Published:** 2026-04-02

**Authors:** Prisca Deviani Pakan, Kiruthika Narayanan, Dennis Chang, Keshav Raj Paudel, Kamal Dua

**Affiliations:** 1 Discipline of Pharmacy, Graduate School of Health, University of Technology Sydney, Ultimo, Sydney, New South Wales, 2007, Australia; 2 Woolcock Institute of Medical Research, Macquarie University, Macquarie Park, Sydney, New South Wales, 2113, Australia; 3 Universitas Nusa Cendana, Department of Pharmacy, Kupang, Nusa Tenggara Timur, 85228, Indonesia; 4 Department of Life Science, University of Technology Sydney, Ultimo, Sydney, New South Wales, 2007, Australia; 5 NICM Health Research Institute & School of Science, Western Sydney University, Westmead, Sydney, New South Wales, 2145, Australia

## ⁯

Respiratory diseases, including asthma, chronic obstructive pulmonary disease (COPD), lung cancer, and pulmonary fibrosis, remain major contributors to global morbidity and mortality (World Health Organization, 2024[7]). In 2022, chronic respiratory diseases ranked third among the leading causes of death worldwide, accounting for close to four million deaths (World Health Organization, 2024[5]). Lung cancer alone represented approximately 11 percent of all newly diagnosed cancers and was responsible for about 18 percent of cancer mortality globally by 2020. The global age-standardized death rate for COPD in 2023 was estimated at 38.7 per 100,000 population, contributing the majority of deaths attributed to chronic respiratory disorders (World Health Organization, 2024[6]).

Despite important advances in pharmacotherapy, many established treatments primarily target symptom control or delay disease progression and are often limited by incomplete efficacy, adverse effects, and substantial inter-patient variability. Current management of asthma and COPD depends largely on inhaled anti-inflammatory agents and bronchodilators, whereas therapies for idiopathic pulmonary fibrosis can slow functional deterioration but do not restore lost capacity. These limitations continue to motivate the search for complementary biological resources that may yield new therapeutic candidates. Respiratory disorders place a disproportionate burden on low- and middle-income settings, where access to standard care may be restricted. Within this context, compounds derived from marine algae are being investigated as potentially accessible adjuncts, provided that their safety, efficacy, and translational relevance can be demonstrated through rigorous study.

Marine algae, encompassing macroalgae and microalgae as well as photosynthetic cyanobacteria, represent a particularly rich reservoir of chemically diverse metabolites. A growing body of literature attributes to these compounds biological activities that intersect with central mechanisms in pulmonary pathology, including airway inflammation, oxidative stress, extracellular matrix remodeling, and dysregulated cell survival. Recent reviews have synthesized evidence suggesting that molecules such as fucoidan, fucoxanthin, astaxanthin, phycocyanin, laminarin, and ulvan demonstrate anti-inflammatory, antioxidant, antifibrotic, and antiproliferative properties in experimental systems relevant to respiratory disease. At the same time, the translational relevance of these findings warrants balanced consideration. Much of the existing evidence has been generated from *in vitro* studies and animal models, which provide valuable mechanistic insight but may not fully represent the clinical complexity of human respiratory diseases (Pakan et al., 2025[4]). In this context, algal metabolites can be considered promising areas for continued research, with future well-designed clinical studies helping to clarify their potential contribution to respiratory care.

A common pathological thread across asthma, COPD, and aspects of pulmonary fibrosis is persistent inflammation coupled with oxidative stress and tissue remodeling. In COPD, chronic airway and parenchymal inflammation involves innate and adaptive immune responses and contributes to progressive airflow limitation. In asthma, guideline-based care increasingly emphasizes inhaled corticosteroid (ICS)-containing regimens for risk reduction and symptom control, reflecting the central role of airway inflammation. Many algae-derived candidates map onto these pathways at a mechanistic level and may complement existing inhaled or systemic therapies rather than act as a standalone treatment, potentially improving outcomes without replacing standard care.

 The various research findings identify fucoidan as a recurring compound of interest in experimental respiratory models, where it has been associated with modulation of NF-κB-mediated inflammatory pathways and cytokine responses. Independent biomedical analyses similarly describe its immunomodulatory and anti-inflammatory properties, while noting limitations related to compositional variability and clinical translation (Apostolova et al., 2020[1]).

In parallel, antioxidant carotenoids and pigment protein complexes, including astaxanthin and phycocyanin, are often discussed as potential countermeasures to oxidative stress and redox imbalance that contribute to airway inflammation and epithelial injury in chronic lung disorders. Experimental studies indicate that these compounds can modulate reactive oxygen species, inflammatory signaling pathways, and cellular defence mechanisms (Pakan et al., 2025[4]).

Within the algae literature, antifibrotic hypotheses often center on interference with profibrotic signaling (including TGF-β-associated pathways), reduced fibroblast activation, and improved redox balance in experimental models. These mechanistic directions are plausible but remain primarily supported by *in vitro* studies and animal models that do not fully recapitulate the heterogeneity and comorbidity. For lung cancer, algae-derived molecules such as fucoxanthin and fucoidan have been associated with antiproliferative and pro-apoptotic effects in cell-based studies, with proposed actions on apoptosis pathways and survival signaling (e.g., PI3K/AKT).

The practical clinical question is more likely whether specific compounds could contribute to adjunctive strategies (for example, through anti-inflammatory or oxidative stress modulation) rather than direct anticancer monotherapy. A central translational consideration in this field relates to the quality of evidence, standardization of materials, and the practical aspects of delivery. The question is not only whether algae-derived compounds demonstrate bioactivity under controlled laboratory conditions, but whether such effects can be reproduced consistently, shown to be safe, and achieved at clinically relevant exposures in the human lung.

One recurring challenge involves variability in the starting material and extraction processes. Species differences, geographic origin, seasonal variation, harvesting conditions, and extraction techniques can all influence chemical composition. As a result, extracts described under similar names may not be pharmacologically equivalent, making direct comparison across studies difficult. Greater standardization and detailed chemical characterization would further help strengthen interpretability and reproducibility.

Bioavailability and pharmacokinetic considerations are also relevant when evaluating marine algae-derived compounds for respiratory applications. Many algal metabolites studied in lung disease models, particularly high-molecular weight polysaccharides such as fucoidan, have physicochemical properties that may limit oral absorption and systemic availability (Hsiao et al., 2021[2]). In several experimental settings, reported biological effects occur at concentrations that may be difficult to replicate *in vivo* through conventional dosing approaches. Formulation strategies, including nanoparticle or liposomal systems, have been explored to improve delivery and tissue exposure of such compounds. However, these approaches introduce additional questions related to stability, reproducibility, scalability, and regulatory assessment that require careful evaluation.

Interpretation of the existing evidence is also shaped by the experimental models used to investigate marine algae in lung disease (Pakan et al., 2025[4]). Much of the current literature relies on *in vitro* systems, rodent models of airway inflammation or bleomycin-induced fibrosis, and established cancer cell lines. These models are useful for examining mechanisms such as modulation of inflammatory signaling, oxidative stress, or fibrotic pathways. At the same time, their capacity to predict clinical outcomes in human respiratory disease is inherently limited (Kokkinis et al., 2024[3]).

Safety considerations of marine algae-derived compounds are equally important. A natural origin does not automatically equate to safety at therapeutic doses, particularly in patients with chronic respiratory diseases who frequently receive multiple concurrent medications. Bioactive constituents isolated from algae, including polysaccharides, carotenoids, and pigment-protein complexes, may exert immunomodulatory or anti-inflammatory effects that are potentially beneficial in inflammatory airway conditions. However, modulation of immune pathways may also have unintended consequences in certain clinical contexts, such as in individuals with active infection, those receiving systemic corticosteroids or other immunosuppressive therapies, or patients with underlying malignancy. In addition, variability in extraction methods, molecular composition, and purity of algal preparations may influence both efficacy and safety profiles. Careful dose-ranging studies, interaction assessments, and standardized characterization of algal products are therefore necessary to clarify safety parameters before broader clinical application can be considered. Taken together, these considerations suggest that the field would benefit from carefully designed translational studies that emphasize standardized compound characterization, clear exposure-response relationships, and clinically meaningful endpoints.

Delivery strategies should also be considered in light of lung biology. For airway diseases, inhaled administration may offer theoretical advantages by enabling localized effects with potentially lower systemic exposure. When appropriate, this approach should be supported by robust aerosol performance testing and inhalation toxicology assessments, extending beyond *in vitro* assays to models that more closely reflect clinical use. Aligning formulation design with pulmonary pharmacology can help clarify feasibility at an early stage.

Marine algae represent a substantial source of bioactive molecules with plausible connections to pathways involved in inflammation, oxidative stress, fibrosis, and disordered cell survival in pulmonary disease. Contemporary syntheses of the literature outline a broad range of candidate compounds and biological mechanisms across asthma, COPD, lung cancer, pulmonary fibrosis, and infectious contexts, while also indicating that much of the available evidence arises from experimental settings.

Within this landscape, algae-derived metabolites can reasonably be considered part of an expanding portfolio of investigational options in respiratory research. Further progress toward clinical relevance will benefit from continued attention to material standardization, pharmacokinetic practicality, delivery strategies, and the development of carefully designed trials that incorporate outcomes meaningful to patients and clinicians.

## Declaration

### Conflict of interest

The authors declare that there are no competing interests.

### Acknowledgments

PDP gratefully acknowledges scholarship support from the Indonesia Endowment Fund for Education (LPDP), Republic of Indonesia, under Grant No. 202402223200571.

### Artificial Intelligence (AI) - assisted technology

AI-assisted technology (only Grammarly) was used exclusively for language refinement, with no role in the development of the manuscript's scientific or original content.
